# Mediastinitis and subcutaneous abscess complicated after EBUS-TBNA of 2R mediastinal lymph node

**DOI:** 10.5578/tt.20239713

**Published:** 2023-09-22

**Authors:** E. Ahmetoğlu, D. Karadoğan, H. Gündoğdu, U. Kostakoğlu, B. Yılmaz Kara, Z. Rakıcı, H. Kazdal, R. Bedir, H. Türüt, Ü. Şahin

**Affiliations:** 1 Department of Chest Diseases, Recep Tayyip Erdoğan University Faculty of Medicine, Rize, Türkiye; 2 Department of Radiology, Recep Tayyip Erdoğan University Faculty of Medicine, Rize, Türkiye; 3 Department of Infectious Diseases and Clinical Microbiology, Faculty of Medicine, Recep Tayyip Erdogan University, Rize, Türkiye; 4 Department of Anesthesiology and Reanimation, Recep Tayyip Erdoğan University Faculty of Medicine, Rize, Türkiye; 5 Department of Pathology, Recep Tayyip Erdoğan University Faculty of Medicine, Rize, Türkiye; 6 Department of Chest Surgery, Recep Tayyip Erdoğan University Faculty of Medicine, Rize, Türkiye

**Keywords:** EBUS, TBNA, complication, mediastinit, subcutaneous abscess

## Abstract

**ABSTRACT:**

**Mediastinitis and subcutaneous abscess complicated after EBUS-TBNA of 2R mediastinal lymph node:**

Endobronchial ultrasound-guided transbronchial needle aspiration (EBUS-TBNA) is a minimally invasive diagnostic tool used for the evaluation of mediastinal lymphadenopathy. It is a safe procedure, but complications such as bleeding and infection may occur. We report a case of a patient who developed a subcutaneous abscess abscess and mediastinitis after EBUS-TBNA. A 75-year-old male with a history of right nephrectomy due to renal cell carcinoma and lung adenocarcinoma history underwent EBUS-TBNA for the evaluation of a right upper paratracheal lymph node. Two weeks after the procedure, the patient presented to the emergency department with skin induration and erythema on the right clavicular area. A non-contrast neck and thorax CT scan was performed, which revealed an extensive subcutaneous abscess on the right clavicular area, extending to the supraclavicular region. The patient was hospitalized, and empirical intravenous antibiotics were initiated due to deep neck infection. Repeated drainage of the subcutaneous abscess was performed. Bacteriologic examination revealed Streptococcus mitis. The patient showed improvement with antibiotic treatment, and a follow-up ultrasound showed a decrease in the size of the abscess and was discharged approximately four weeks after hospitalization. Although very rare, serious infectious complications may develop after EBUS-TBNA, and our case report is an important example regarding its management process.

## Abstract (tr)

**2R mediastinal lenf nodunun EBUS-TBİA’sı sonrası komplike mediastinit ve subkütan apse:**

Endobronşiyal ultrasonografi kılavuzluğunda transbronşiyal iğne aspirasyonu (EBUS-TBİA), mediastinal lenfadenopatilerin değerlendirilmesinde kullanılan minimal invaziv bir tanı aracıdır. Güvenli bir işlem olmakla beraber, kanama ve enfeksiyon gibi komplikasyonlar ortaya çıkabilir. EBUS-TBİA işlemine bağlı derin boyun enfeksiyonu, cilt altı apse ve mediastinit gelişen bir hastayı sunmaktayız. Renal hücreli karsinom sebebiyle sağ nefrektomi ve akciğer adenokarsinomu öyküsü olan 75 yaşında erkek hastaya sağ üst paratrakeal lenf nodunun incelenmesi için EBUS-TBİA işlemi uygulandı. Hasta işlemden iki hafta sonra sağ klaviküler bölgede şişlik ve kızarıklık sebebiyle acil servise başvurdu. Çekilen kontrastsız boyun ve toraks bilgisayarlı tomografide sağ klaviküler bölgeden supraklaviküler bölgeye uzanan geniş cilt altı apsesi gözlendi. Hasta derin boyun enfeksiyonu sebebiyle interne edildi ve ampirik intravenöz antibiyoterapi başlandı. Cilt altı apsesi birçok kez drene edildi. Alınan drenaj materyalinde Streptococcus mitis üredi. Hasta antibiyoterapiden klinik olarak fayda gördü ve yapılan ultrasonda apsenin boyutunda azalma görüldü. Hasta internasyonundan dört hafta sonra taburcu edildi. EBUS-TBİA’dan sonra ciddi enfeksiyöz komplikasyonlar çok nadir olsa da gelişebilmektedir. Vaka sunumumuz bunun yönetimi ile ilgili önemli bir örnektir.

## INTRODUCTION

Endobronchial ultrasound-guided transbronchial needle aspiration (EBUS-TBNA) is a diagnostic method for mediastinal and hilar lymphadenopathy ( 1 ). It is mainly used in nodal staging of lung cancer patients. EBUS-TBNA is also used as a diagnostic procedure in cases of mediastinal lymphadenopathy such as patients with sarcoidosis, tuberculosis and lymphomas. The cumulative sensitivity of EBUS-TBNA in lymph node stages of lung cancer is 88% -93% ( 2 ). Since its proposal in 2004 ( 1 ), EBUS has become an important diagnostic tool in the evaluation of intrathoracic lymph nodes and other structures adjacent to the trachea and has become an effective alternative to mediastinoscopy, which is a surgical intervention with relatively higher risks and complications, as its diagnostic accuracy is at least equivalent to that of mediastinoscopy and much greater for some lymph node stations ( 3 ), and a recently published meta-analysis has noted no important complications among >1.500 completed procedures ( 4 ).

Complications from EBUS-TBNA are essentially those associated with both bronchoscopy and TBNA ( 5 ). According to a national survey conducted by the Japanese Society of Respiratory Endoscopy, the infectious complication rate for EBUS-TBNA was 0.19% ( 6 ). In one study of 3123 patients who underwent EBUS-TBNA, the incidence of infectious complications occurred in 0.16% ( 7 ). Serious complications such as mediastinal abscess, pneumothorax, empyema, pericarditis, sepsis, and intracavitary hematoma have been described in case reports ( 6 ). We should note that most of the studies on infectious complications and other serious complications are case reports, and if there are large studies, they are mostly short-term and do not separate specific complications such as infectious complications, but rather focus on the overall complications ( 8 ). The aim of the present case report is to report on rare complications of EBUS-TBNA that have been successfully treated by repeated drainage and antibacterial therapies.

## CASE REPORT

**Figure 1:**
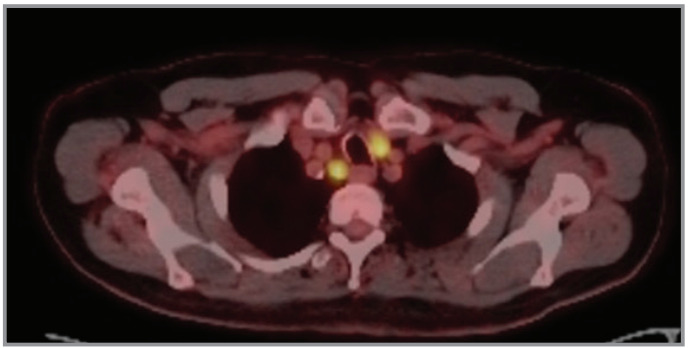
PET-CT revealed hypermetabolic lymph node in the right upper paratracheal area (2R station).

A 75-year-old male with a history of right nephrectomy due to renal cell carcinoma was diagnosed with lung adenocarcinoma two years ago (T2aN2M0 moderately differentiated adenocarcinoma in the left upper lobe). The patient underwent chemotherapy and radiotherapy. He had chronic kidney disease, chronic obstructive pulmonary disease (GOLD Category B), coronary artery disease, and was a former smoker with 75 pack-years smoking history. In November 2022, thoracic CT was studied for follow-up purposes, revealing a lymph node in the right upper paratracheal area measuring 11 mm in short size. Compared to the previous review, the size had increased. In the left hilum, there was an irregularly shaped soft tissue surrounding the main bronchi. PET-CT revealed a hypermetabolic lymph node in the right upper paratracheal area (2R station). Compared to the F-18 FDG PET/CT audit dated 09.06.2022, the dimensions of the lymph node in the right upper paratracheal area increased markedly, and it acquired pronounced hypermetabolism. It has been thought that it may be metastatic in the light of the images through the evaluation of the multidisciplinary team that included pulmonologist, thoracic surgeons, oncologists, nuclear medicine specialist and radiologist, and tissue sampling from the right upper lymph node was recommended ( Figure 1 ).

**Figure 2:**
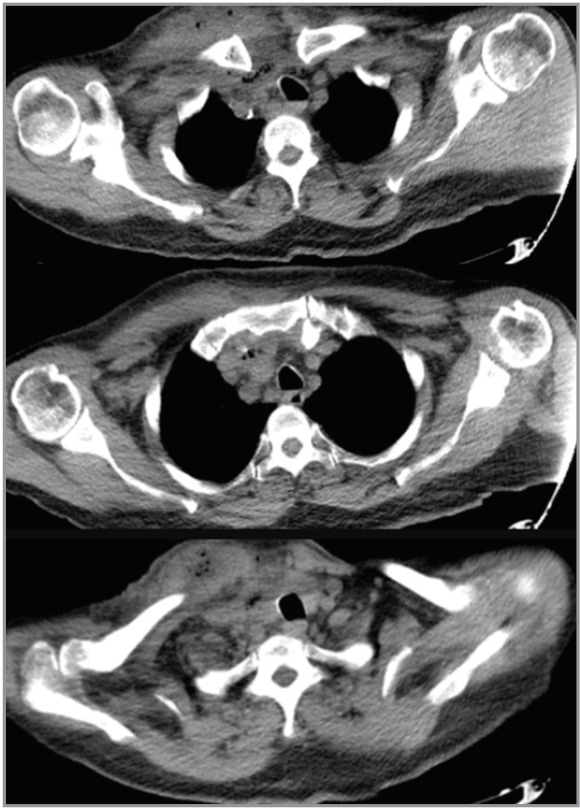
Mediastinal section of a thoracic computed tomography of the subject with an extensive subcutaneous abscess on the right clavicular area, extending to the suprascapular region with air densities and soft tissue density and free air densities compatible with pneumomediastinum was observed.

We performed EBUS-TBNA for pathological evaluation of the lesion using a convex type-EBUS scope (Fujifilm, Japan), and the enlarged node was identified. Multiple needle biopsies were taken from the right mediastinum 2R station (11 mm) three times, 10-15 suctions were performed each time, the first biopsy was also done with clinodevice needle (22-Gauge) and the other two biopsy was done with Cook needle (22-Gauge). It should be noted that the procedure was under the supervision of an anesthesiologist. Bispectral index monitoring was used in the administration of anesthesia to create a deep level of sedation, and the patient was administered with intermittent propofol in addition to the initial intravenous 2 mg midazolam and 50 microgram fentanyl with a BIS value of 60-70. After discharge, the patient applied to the emergency department and neurosurgery clinic with complaints of diffuse pain in the neck within two weeks, but no results were obtained. Two weeks after the procedure, the patient was presented to the infection disease outpatient clinic due to skin induration and erythema on the right clavicular area. Physical examination revealed no crepitation and subcutaneous emphysema. Laboratory examination comprising routine blood and biochemical tests was normal except for C-reactive protein (CRP) level (275 mg/dL), creatinine level (3.82 mg/dL), and glomerular filtration rate eGFR (14 mL/min). Ultrasound of the neck showed a dense collection area of approximately 2.5 cm in diameter in the right supraclavicular region, suggesting an abscess. A non-contrast neck and thorax CT was performed, which revealed an extensive subcutaneous abscess on the right clavicular area, extending to the supraclavicular region with air densities that were also observed in the collection area. Within the anterior mediastinum, soft tissue density and free air densities compatible with pneumomediastinum were observed ( Figure 2 ).

The patient was hospitalized to the pulmonology inpatient clinic, and empirical antibiotic treatment for suspected deep neck infection or mediastinitis was started. Upon the growth of hyperemia and swelling on the skin, infectious diseases and interventional radiology were contacted. Interventional radiology aspirated 30 cc abscess from the dense collection area in the subcutaneous soft tissue at the right upper thoracic level of the patient. A 10F drainage catheter was placed in this localization. The abscess culture was examined, and Streptococcus mitis was detected from the drainage specimen culture. The patient showed improvement with the treatment, and a follow-up ultrasound showed a decrease in the size of the abscess. On the 26th day of empirical Tigecycline and Ciprofloxacin treatment, control was performed with superficial USG, and the patient was discharged afterwards.

## DISCUSSION

Endobronchial ultrasound-guided transbronchial needle aspiration (EBUS-TBNA) is an important diagnostic procedure to assess mediastinal and hilar lymphadenopathy ( 1 ). In general, EBUS-TBNA is the preferred first-step procedure for sampling large, centrally located tumors and for suspicious nodal involvement in the mediastinum as well as hilar lymph nodes (2R, 2L, 3p, 4R, 4L, 7, 10R, 10L, 11R, 11L) ( 5 ). Importantly, although diagnosis and staging can be performed simultaneously, they can also be performed sequentially. Suspicious nodes include those that are enlarged by computed tomography or metabolically active by positron emission tomography (PET). The preference for EBUS is based upon the reported high sensitivity of EBUS-TBNA to stage and diagnose non-small cell lung cancer (NSCLC) in this setting and its ability to access more nodal stations than the traditional gold standard, cervical mediastinoscopy. Multiple needles passed by an experienced operator and rapid on-site cytologic evaluation are suggested to increase the diagnostic yield of EBUS-TBNA samples and provide samples that are adequate for genetic analysis ( 5 ). Numerous publications about mediastinoscopy described minor in 4.7% to 5.4% of patients ( 7 , 9 ), there is a much higher incidence of major complications with mediastinoscopy in comparison with EBUS-TBNA ( 9 ). According to a systematic review, EBUS-TBNA can rarely lead to serious complications such as respiratory failure, bleeding and infection ( 8 ). Infection is the most common complication of EBUS-TBNA and can be life-threatening, requiring long hospitalization and causing high costs financially ( 4 , 7 ). Due to the rarity of infectious complications of EBUS-TBNA, we know little about the risk factors for these complications as most of the studies are case studies, and large studies are conducted in the form of a questionnaire survey focusing on EBUS complications in general ( 7 , 8 ). We should note that the most important advantage of EBUS-TBNA is the rarity of its complications. In previous studies, infectious complications ranged from 0.19% to 0.54% ( 8 ).

In our study, Streptococcus mitis growths were detected on abscess culture. The hypothesized mechanism is caused by infection of the mediastinal tissue with oropharyngeal bacteria during the transbronchial passage of the needle ( 8 ). The needle is supposed to be sterile. Contamination of the sterile needle may occur as it passes through the working channel of the bronchoscope or within the airway during biopsy, and it is suggested that the risk of contamination is higher if a bronchoscope is used to clean airway secretions by suction ( 10 ). In our case, a 75-year-old male patient with a history of cancerous tumor who received chemotherapy underwent EBUS-TBNA using convex-probe bronchoscopes (Fujifilm, Japan). The scope was inserted through the oral route, in supine position and deep sedation where intermittent propofol in addition to initial intravenous 2 mg midazolam and 50 microgram fentanyl was administered to the patient. 22-gauge needles were used for the procedure by the hands of an expert specialist, a multi-needle biopsy was taken from the right mediastinum station 2R (11 mm) three times (10-15 suctions were performed each time). After the procedure, the patient developed a deep neck abscess and mediastinitis, which is a rare complication.

A study that looked at possible complications of EBUS-TBNA included infectious complications and associated risk factors, such as immunosuppressive patients and those taking immunosuppressants, chemotherapy or chronic corticosteroids, or in patients with cystic target lesion or if more than ten punctures were made for the same lesion during the procedure, or who had chronic bronchial colonization ( 9 ). All of these mentioned above are potential risk factors. According to this study, we should also note that other previous studies have also suggested that the presence of necrosis in the target lesion during the EBUS-TBNA procedure is a risk factor for infection and hypothesized this due to vascular deficiency ( 8 , 9 ). Therefore, many experts recommend avoiding dead or cystic lesions ( 11 ). We did not detect necrosis by computed tomography or EBUS in any of the lymph nodes/lesions in our patient; however, the patient had a history of tumor and underwent chemotherapy and radiotherapy months ago, with multiple diseases such as renal insufficiency, and it must be emphasized that during the intervention, more than 10 punctures were performed, which are all suspected as risk factors.

In total, the number of examined lesions, the number of EBUS needles used, and the number of passes per patient have each been suspected as risk factors of infectious complications. However, none of the procedure details were statistically associated with infectious complications ( 8 ). In order to investigate potential risk factors, in one study of the patients who received EBUS-TBNA and were followed up for at least two months, the group that experienced postprocedure mixed infections did not have any patients using steroids or immunosuppressants in the infection group, but it was noted that there were more of diabetic patients in the infection group compared to the control group, but this was not statistically significant ( 8 ). In general, diabetes or immunodeficiency status were proposed risk factors without clear statistical evidence since it is believed that patients with these conditions have an increased tendency to infection, and in the same study, the focus was on the number of lesions examined, and the number of EBUS needles used, and the number of passes for each patient, and no statistical indications were obtained to prove that they are risk factors for infectious complications ( 8 ). To summarize the study, infectious complications occurred in 33 patients (0.48%) of the total 6826 patients who were EBUS-TBNA administered and followed, with an average incidence of eight days after the procedure ( 8 , 9 ). The incidences of complications may vary depending on several factors ( 7 , 9 ). First, follow-up of the patients to detect possible complications is not often a routine procedure, and lack of documentation is also an important factor, especially for minor complications. Second, the definition of complications and their severity can affect the rates and descriptions of the complications ( 7 ). Considering the investigations and case reports reported in the literature, infectious complications constitute a significant proportion of serious complications. Prolonged hospitalization was required in subjects with serious complications like our case. Similarly, Asano et al. have reported prolonged hospitalization in 14 (15.6%) of 90 cases with complications of EBUS-TBNA of a total of 7345 cases ( 6 ).

In one study of 489 patients diagnosed with EBUS-TBNA, 30 patients were from the infection group, and 459 were from the controls. The infection group (n= 30) consisted of pneumonia (n= 18) and mediastinum infections (n= 12). The median of days to start antibiotic therapy after the procedure was seven. Compared to the pneumonia group (median, five days), it took longer to recognize the occurrence of infectious complications in the mediastinal infection group (median, nine days). Five patients received an intervention to control the infection, either surgically (n= 3) or percutaneous drainage (n= 2), all from the mediastinal infection group. The total duration of antibiotic treatment was longer in the mediastinal infection group (median, 27 days) than in the pneumonia group (median, 13 days) ( 8 ). The use of prophylactic antibiotics is unproven. The guidelines of the European Society of Gastrointestinal Endoscopy recommend the use of prophylactic antibiotics, but this has not been approved by the American College of Chest Physicians or the American Society of Bronchopulmonary Diseases because it may not penetrate vascular structures such as large necrotic lymph nodes, which can be the desired targets of diagnosis ( 8 ).

## CONCLUSION

Infectious complications after EBUS-TBNA are seen rarely, and they are more common especially in patients with risk factors for infection. We presented a case who developed complications in the form of mediastinitis and deep neck infection due to the EBUS-TBNA procedure. EBUS decisions should be made by a multidisciplinary team, and patients should be informed about possible complications of the procedure.

## CONFLICT of INTEREST

The authors have no conflict of interest to declare.

## AUTHORSHIP CONTRIBUTIONS

Concept/Design: EA, DK, UK, ÜŞ, BYK

Analysis/Interpretation: EA, DK, UK, HG

Data acqusition: EA, DK, ZR

Writing: EA, ZR

Clinical Revision: ÜŞ, HT, RB, HK, BYK

Final Approval: All of authors
